# Empirical validation of the information-motivation-behavioral skills model of gestational weight management behavior: a framework for intervention

**DOI:** 10.1186/s12889-023-15067-2

**Published:** 2023-01-18

**Authors:** Hua You, Yuan-Yuan Wang, Chi Zhang, Anita Nyarkoa Walker, Jin-Jin Ge, Shi-Qi Zhao, Xue-Qing Peng

**Affiliations:** 1 School of Public Health, Nanjing Medical University, Nanjing, China; 2 Institute of Healthy Jiangsu Development, Nanjing Medical University, Nanjing, China; 3 School of Nursing, Nanjing Medical University, Nanjing, China; 4 Jiangsu Health Development Research Center, Nanjing, China; 5 Chengdu Center for Disease Control and Prevention, 4 Longxiang Road, Wuhou District, Chengdu, 610041 China

**Keywords:** Gestational weight management, Gestational weight gain, Information-Motivation-Behavioral skills model, Behavioral model, Structural equation modelling

## Abstract

**Background:**

Unhealthy gestational weight gain is a modifiable risk factor for adverse maternal and child health. Appropriate and effective intervention strategies that focus on behavioral change or maintenance are critical in weight management during pregnancy. Our aim was to uncover the influencing factors and psychosocial mechanisms of gestational weight control behavior, and to construct a behavioral model suitable for intervention based on Information-Motivation-Behavioral skills (IMB) model.

**Methods:**

A sample of 559 pregnant women from a municipal maternal and child healthcare facility in Jiangsu Province, China was enrolled in this cross-sectional empirical study. Partial least square structural equation modelling was used to verify the hypothesized model, and post hoc analyses was used to test the effect of parity and pre-pregnancy BMI on the model.

**Results:**

The IMB model elements can predict gestational weight management (GWM) behavior well, with information being the most influential factor. As predicted, information affects GWM directly (β = 0.325, *p* < 0.05) and indirectly (β = 0.054, *p* < 0.05) through behavioral skills. Likewise, motivation has direct (β = 0.461, *p* < 0.05) effects on GWM, and has indirect (β = 0.071, *p* < 0.05) effects through behavioral skills. Behavioral skills have a direct impact (β = 0.154, *p* < 0.05). The model had a goodness of fit (GOF = 0.421) and was robust when tested in subgroups of different parity or pre-pregnancy BMI.

**Conclusion:**

Findings from this study supported the predictions of the IMB model for GWM behavior, and identified its modifiable determinants. The tested behavior model for GWM can serve as a new validated intervention strategy in weight management among pregnant women.

## Background

Gestational weight gain is a public health issue that deserves global attention. An increasing number of evidences has suggested that gestational weight gain above or below the recommended weight gain range is associated with many pregnancy-related adverse health outcomes [1, 2]. Maintaining a healthy diet and adequate amount of exercise are effective strategies to reduce excessive weight gain during pregnancy [3]. Nevertheless, globally, various countries and regions have reported unreasonable diet and insufficient exercise problems of pregnant women [4, 5]. These unhealthy behaviors can lead to serious adverse pregnancy outcomes [6, 7], causing a heavy burden on individuals, families, and the society.

Excessive weight gain during pregnancy is an important issue of worldwide concern. More than 50% of American pregnant women and nearly 40% to 50% of Chinese pregnant women have been reported to gain excessive weight during pregnancy [8, 9]. Meanwhile, insufficient weight gain during pregnancy is also prevalent worldwide [10]. Also, increase in insufficient or excessive gestational weight gain during the COVID-19 pandemic has been observed [11], as they develop emotional eating with insufficient physical activity [12]. Hence, strengthening the weight management intervention for pregnant women is a key issue.

Different health behavior theories have been combined to explain and intervene in weight management during pregnancy, such as planned behavior theory [13], protection motivation theory [14], social cognitive theory [15], and PRECEDE-PROCEED model [16], etc. Multiple studies have found theory-based interventions were more effective in affecting and sustaining behavioral changes than non-theory-based interventions [14, 17]. However, previous meta-analyses found that the results of the two types of interventions were similar, and even the effect of theory-based interventions was worse [18, 19]. This might be ascribed to the limited suitability of most existing intervention theories for weight management during pregnancy, prompting us to explore more appropriate behavioral models.

**Fig. 1:**
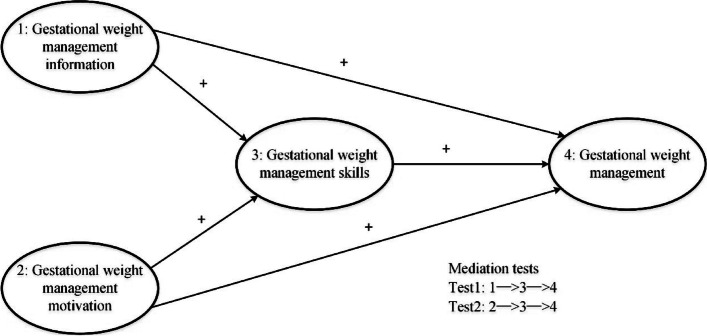
Hypothesized structural equation model with two mediation tests. Ellipses represent the latent variables. The “ + ” between ellipses represent the hypothetical path relationship between the key variables

In this study, we applied the Information-Motivation-Behavior skills(IMB) model proposed by Fisher et al. to construct our hypothesized model (Fig. 1). The IMB model elucidated that the generation and maintenance of behavior is determined by three factors: information, motivation, and behavioral skills, and there are complex pathways between these factors [20]. It has empirically applied in the interpretation and intervention of various health related behaviors [21–23]. Using a structural equation modelling(SEM) approach, we aimed to: (1) test whether our hypothesized model holds, that gestational weight management(GWM) is impacted by information, motivation and behavioral skills and (2) assess the direct, indirect, and overall effects between these three factors on GWM. Specifically, we found that parity and pre-pregnancy body mass index(BMI) were the frequently mentioned factors that associated with gestational weight gain [24, 25], which may interfere with our model. Hence, we considered parity and pre-pregnancy BMI for post hoc analysis to test the model’s robustness.

## Methods

### Study design and participants

To verify our hypothesized model, we adopted purpose sampling to select the survey objects. In this purpose sampling, we focused on the individuals who can provide the adequate information required to achieve the goal of the research [26]. Our survey site was Changzhou Maternity and Child Health Care Hospital, Jiangsu Province, China. It is a municipal public specialized medical institution that undertakes more than half of the annual delivery volume in the city. We invited pregnant women from obstetrics clinic of the hospital who met the inclusion criteria to participate. The inclusion criteria were (1) not less than 18 years old; (2) the duration of pregnancy (pregnancy period >  = 14 weeks); (3) singleton pregnancy; (4) without medical complications such as heart, liver and kidney, and diseases like primary hypertension and diabetes; (5) able to communicate and can self-report independently.

We recruited four medical staff who had received unified investigation training as investigators. The data was collected by a face-to-face questionnaire investigation. The survey took place from September 2020 to October 2020. Following the rule of tenfold scale item numbers and considering 15% of invalid questionnaires, we determined that the minimum sample size for this study was 435 (since the number of scale items in this study was 37). A total of 585 pregnant women were recruited, out of which the questionnaires of 26 pregnant women were eliminated due to the omission of key information. Finally, the questionnaire of 559 pregnant women were included for analysis. A verbal consent was obtained from the respondents before the investigation. The investigation lasted for about 15–20 min. After the questionnaire investigation, each pregnant woman received a postpartum rehabilitation service voucher provided by the hospital as a gift. This research was approved by the Ethics Review Committee of Nanjing Medical University (No. (2020)63).

### Measures

#### Questionnaires based on the IMB model

This part is about information, motivation, and behavioral skills related to GWM as defined based on the IMB model. We measured it with three self-designed scales, and all the items were designed by learning the operational definitions of IMB theory and reviewing similar works on gestational weight management [27–29]. For the content of these self-designed scale items, we have invited experts to evaluate and conducted a small sample pilot test. All items were measured on a 5-point scale(1 = completely unknown/disagree to 5 = completely understood/agree). ①The information subscale(included 5 items, Cronbach's α = 0.809). This was used to measure the pregnant women's knowledge of GWM, such as "Adult’s body mass index standards and how to calculate it". The higher the score, the better the understanding of the information. ②The motivation subscale(included 5 items, Cronbach's *α* = 0.847). This was also used to measure pregnant women’s motivation for GWM, including personal motivation and social motivation. For instance, "I think it is necessary to manage weight during pregnancy" and "The medical staffs encouraged me to manage weight during pregnancy". The higher the score, the higher the motivation. ③The behavioral skills subscale (included 5 items, Cronbach's *α* = 0.840). It measured the pregnant women's mastery of the skills required for weight management during pregnancy, such as "I have the ability to assess whether my amount of gestational weight gain in the weeks of pregnancy is reasonable". The higher the score, the higher the mastery of behavioral skills. For this whole scale, the Cronbach’s *α* = 0.894, the Kaiser–Meyer–Olkin (KMO) value = 0.888, and the *p* value of Bartlett’s test < 0.001. Those all indicated the reliability and validity of this scale were acceptable.

#### Gestational weight management behavior

The pregnancy weight management strategy scale compiled by Yan et al. was used to assess weight management behavior during pregnancy [30]. There were 22 items involved a measure of diet, physical activity, self-monitoring, and self-regulation. All the items were evaluated on a 5-point scale (1 = never to 5 = always). We use the total scores of each questionnaire to represent the respondent's weight management status. The total score ranged from 22 to 110. A higher total score implies better behaviors in GWM. The Cronbach's α of the modified scale was 0.823, the KMO was 0.825, and the Bartlett’s test *p* value < 0.001. These values suggested that this modified scale has certain reliability and validity.

#### Maternal demographic and biomedical characteristics

Participants also reported their birth date, occupation, educational level, average annual family income, parity, and their pre-pregnancy height and weight.

### Statistical analysis

We used SPSS23.0 software to describe the participants’ basic characteristics, the scores of each dimension of the IMB model, and the GWM score. Then, we evaluated the bivariate correlation between the weight management related IMB model factors and GWM behavior. Structural equation modeling was observed to be a very robust and powerful statistical tool in many disciplines [31], so we adopted the SEM method to assess the hypothesized model. Before analysis, the normality of the data was checked on the basis of kurtosis and skewness. Some items had peaks greater than the suggested criterion of 0.3, suggesting that the data were not normally distributed [32]. Partial least square structural equation modeling showed better performance in non-normally distributed data and was suitable for model validation of theoretical development [33], so we utilized SmartPLS3.3.1 software for analysis.

To perform SEM, in the first stage, we assessed the measurement model’s reliability and validity of each dimension of the gestational weight management IMB model. The factor loading(generally should be > 0.7, but Hair et al. suggested that 0.4 ~ 0.7 was also acceptable under the condition that the average variance extracted(AVE) was > 0.5 [34]). Cronbach's α(> 0.7), composite reliability(CR, > 0.7) and AVE(> 0.5) were used to assess reliability. Fornell-Larcker standard and Heterotrait-Monotrait (HTMT, < 0.85) were used to assess validity [35, 36]. The second stage was to evaluate the structural model to test the relationship between the various dimensions. We conducted 5000 bootstrapping algorithms to assess the significance of the path coefficients, and evaluate the structural model’s validity according to the suggested values of R^2^, Q^2^ and goodness of fit(GOF) [35, 37].

To test the robustness of the model, we performed a post hoc analysis using t-test (two-tailed) and partial least square multi-group analysis(PLS-MGA) function, mainly to test whether the model was affected by the parity(0 = primipara; 1 = multipara) and pre-pregnancy BMI(0 = normal weight; 1 = underweight; 2 = overweight & obesity). The significance level was two-sided α = 0.05.

## Results

### Descriptive analysis for the basic characteristics of respondents

In Table 1, participant’s average age was 29.2(SD = 4.3) years old; 39.9% of pregnant women had college educational level or above; 58.3% were employed; 47.2% had an average annual family income of more than 120,000(CNY). The average pre-pregnancy BMI was 22.4 kg/m^2^(SD = 3.5, range15.6–40.6), which accounted for 54.9% of pregnant women’s pre-pregnancy BMI within the normal range; primipara and multipara accounted for 46.0% and 54.0%, respectively. The motivation score was at an upper-middle level, and the information and behavioral skills scores were at a middle level. GWM score was at a moderate level(57.8 ± 12.6).

**Table 1:** Basic characteristics of pregnant women (*N* = 559)

Variables	N/Mean	Percentage/SD
**Demographic characteristics**		
Age, y	29.2	4.3
Education		
Primary school	2	0.4
Junior high school	113	20.2
High School/Technical School	36	6.4
Technical secondary school/technical	39	7.0
Junior college	146	26.1
University and above	223	39.9
Occupation		
Employee	326	58.3
Unemployed	125	22.4
Freelance	58	10.4
Self-employed	44	7.9
Farmer	1	0.2
Other	5	0.9
Annual household income, CNY		
< 30,000	8	1.4
30,000—50,000	22	3.9
50,001—70,000	32	5.7
70,001—100,000	113	20.2
100,001—120,000	120	21.5
> 120,000	264	47.2
**Biomedical characteristics**		
Pre-pregnancy BMI (kg/m^2^)^a^	22.4	3.5
Underweight	45	8.1
Normal weight	307	54.9
Overweight	113	20.2
Obesity	94	16.8
Parity		
Primipara	257	46.0
Multipara	302	54.0
**IMB factors**		
Information	15.0	3.4
Motivation	19.5	4.2
Behavioral skill	16.8	4.2
**Gestational weight management**		
Weight management	57.8	12.6

^a^The BMI category was classified using the WHO recommended classification criteria for Asian populations

### Bivariate relationship among key variables

The binary correlation between the key variables in the path model showed that GWM positively correlated with information(*r* = 0.471), motivation(*r* = 0.389) and behavioral skills(*r* = 0.419), *p* < 0.05. Behavioral skill had a positive correlation information(*r* = 0.526) and motivation(*r* = 0.595), *p* < 0.05 (Table 2).

**Table 2:** Bivariate correlation analysis between key variables

Variables	Information	Motivation	Behavioral skills	Weight management
Information	—	0.393^**^	0.526^**^	0.471^**^
Motivation		—	0.595^**^	0.389^**^
Behavioral skills			—	0.419^**^
Weight management				—

^**^p < 0.01

### Measurement model results

The factor loadings ranged from 0.668 to 0.852. The Cronbach's ɑ range for each dimension was 0.837 ~ 0.849, and the CR values were between 0.885 and 0.892, indicating a sufficient internal consistency. The AVE values ranged from 0.609 to 0.624, indicating a good convergence validity (Table 3).

**Table 3:** Reliability and convergence validity of the measurement model

Constructs	Item labels	Mean	SD	Loadings	Cronbach's ɑ	CR	AVE
Information (In)	In1	3.13	0.70	0.797	0.837	0.885	0.609
	In2	3.00	0.73	0.803			
	In3	3.16	0.79	0.826			
	In4	3.28	0.89	0.796			
	In5	2.47	1.24	0.668			
Motivation (Mo)	Mo1	4.04	0.98	0.781	0.849	0.892	0.624
	Mo2	4.05	0.97	0.737			
	Mo3	3.93	1.07	0.807			
	Mo4	3.58	1.14	0.848			
	Mo5	3.92	1.11	0.772			
Behavioral skills (BS)	BS1	3.32	1.05	0.781	0.842	0.888	0.615
	BS2	3.22	1.06	0.821			
	BS3	3.33	1.05	0.852			
	BS4	3.58	1.04	0.785			
	BS5	3.32	1.12	0.669			

The AVE root values were bigger than the correlation coefficients between the dimensions, and the HTMT values between the dimensions were all < 0.85, indicating that the measurement models had a good discriminative validity (Table 4).

**Table 4:** Differential validity of the measurement model

**Fornell-Larcker Criterion**	**Weight management**	**Behavioral skills**	**Information**	**Motivation**
Weight management	**1.000**			
Behavioral skills	0.429	**0.784**		
Information	0.473	0.526	**0.780**	
Motivation	0.392	0.596	0.387	**0.790**
**Heterotrait-Monotrait (HTMT)**	**Weight management**	**Behavioral skills**	**Information**	**Motivation**
Weight management				
Behavioral skills	0.466			
Information	0.518	0.622		
Motivation	0.422	0.701	0.460	

### Structural model results

The R^2^ of behavioral skills and GWM behavior were 0.458 and 0.288, respectively, indicating a higher predictive power. The Q^2^ values of behavioral skills and GWM were 0.277 and 0.280, respectively, indicating the predictive correlation of the structural model. The model had a good degree of fit(GOF = 0.421).

**Fig. 2:**
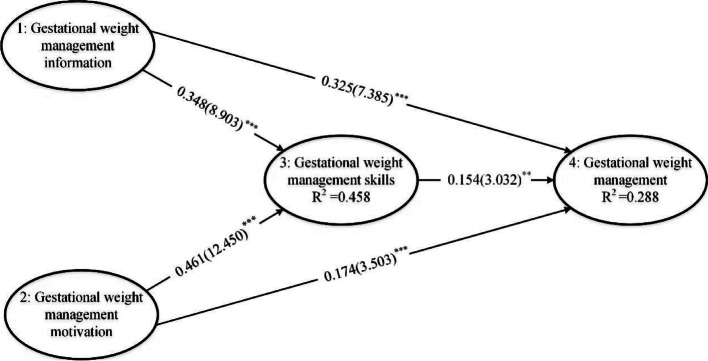
Path relationship diagram. Ellipses represent the latent variables. Arrows between ellipses represent standardized regression path coefficients. The values in brackets are the t statistics. **p < 0.01, ***p < 0.001

Figure 2 showed the standardized path coefficients of the hypothesized model. Information, motivation, and behavioral skills all had an impact on GWM. In Table 5, the results showed that information directly and positively affected behavioral skills(β = 0.348) and GWM behavior(β = 0.325), and indirectly affected GWM behavior(β = 0.054) through behavioral skills, *p* < 0.001. Motivation can have a direct positive effect on behavioral skills(b = 0.461) and weight management behaviors during pregnancy(β = 0.174), and an indirect positive effect on GWM behaviors through behavioral skills(β = 0.071), *p* < 0.001. Behavioral skills only had a direct positive effect on weight management behavior(β = 0.154, *p* < 0.01). For the total effects, information was the most important factor affecting GWM.

**Table 5:** Decomposition of the effect of IMB factors on GWM

Path	Direct effect	Indirect effect	Total effect
Information—> Behavioral skills	0.348^***^	-	0.348^***^
Information—> Weight management	0.325^***^	0.054^**^	0.378^***^
Motivation—> Behavioral skills	0.461^***^	-	0.461^***^
Motivation—> Weight management	0.174^**^	0.071^**^	0.245^***^
Behavioral skills—> Weight management	0.154^**^	-	0.154^**^

^**^p < 0.01

^***^p < 0.001

### Post hoc analysis: parity and pre-pregnancy BMI

In Table 6, there were no differences in the scores of each construct between the different parities. And there was no difference in the scores of all the variables between the underweight and normal weight group. But compared with the pre-pregnancy overweight and obesity group, the normal weight group had more behavioral skills, *p* < 0.01. The PLS-MGA results in Table 7 showed that there were no significant differences in all path relationships between different parities and pre-pregnancy BMI groups, indicating that parity and pre-pregnancy BMI had no effect on the model.

**Table 6:** Differences in IMB factors and GWM behaviors of pregnant women with different parity and pre-pregnancy BMI

**Factors**		**Information**	**Motivation**	**Behavioral skills**	**Weight management**
Parity	Primipara (*n* = 257), mean (SD)	3.02(0.69)	3.91(0.85)	3.35(0.80)	2.62(0.59)
	Multipara (*n* = 302), mean (SD)	3.00(0.66)	3.90(0.81)	3.36(0.86)	2.63(0.56)
	t test (df*)*	0.44(557)	0.08(557)	-0.18(557)	-0.28(557)
	*p* value	0.662	0.940	0.861	0.777
Pre-pregnancy BMI	Normal weight (*n* = 307), mean (SD)	3.02(0.70)	3.92(0.85)	3.41(0.83)	2.65(0.58)
	Underweight (*n* = 45), mean (SD)	3.16(0.70)	3.99(0.87)	3.56(0.86)	2.57(0.57)
	t test (df*)*	-1.26(350)	-0.48(350)	-1.12(350)	0.92(350)
	*p* value	0.210	0.630	0.263	0.357
	Normal weight (*n* = 307), mean (SD)	3.02(0.70)	3.92(0.85)	3.41(0.83)	2.65(0.58)
	Overweight &obesity (*n* = 203), mean (SD)	2.96(0.62)	3.86(0.79)	3.22(0.81)	2.59(0.55)
	t test (df*)*	0.90(508)	0.88(508)	2.61(508)	1.19(508)
	*p* value	0.370	0.378	0.009	0.234

**Table 7:** Multi-group analysis of parity and pre-pregnancy BMI

Path relationships	Parity		Pre-pregnancy BMI			
	Path difference	*p* value^a^	Path difference	*p* value^b^	Path difference	*p* value^c^
In—> GWM	0.02	0.859	0.02	0.870	-0.21	0.112
In—> BS	-0.02	0.784	-0.14	0.098	-0.11	0.308
In—> BS—> GWM	0.06	0.100	-0.01	0.924	0.05	0.459
Mo—> GWM	-0.10	0.312	0.16	0.162	-0.002	0.980
Mo—> BS	0.03	0.681	0.08	0.294	-0.05	0.606
Mo—> BS—> GWM	0.09	0.057	0.03	0.526	0.09	0.387
BS—> GWM	0.19	0.062	0.04	0.715	0.18	0.342

*In* Information, *Mo* Motivation, *BS* Behavioral skills, *GWM* Gestational weight management

^a^Multipara *vs.* Primipara

^b^Overweight(&obesity) *vs.* Normal weight

^c^Underweight *vs.* Normal weight

## Discussion

Based on the IMB model, we constructed a behavioral model for gestational weight management, and conducted an empirical research among Chinese pregnant women. Our results supported all the hypotheses, confirming that pregnant women’s weight management behavior is affected by information, motivation and behavioral skills factors. The interpretation of GWM behaviors by the model was good (*R*^2^ = 0.288) [37]. We also performed a post-hoc analysis to test the robustness of the model. And the results suggested that our model were applicable to explaining pregnant women's GWM behaviors, both in the multiparous and primiparous, and no matter how their pre-pregnancy BMI status was.

Consistent with the original IMB model, our results showed that information can directly affect GWM behavior, and indirectly affect through behavioral skills. Information (knowledge that highly relate with behaviors) is an essential prerequisite for reducing risky behavior, and it can also stimulate behavioral skills to change and maintain behavior [20]. Our results confirmed this, indicating the multiple roles of information in influencing healthy behaviors. Previous qualitative studies found that pregnant women were not aware of the adverse effects of overweight or obesity during pregnancy, and misunderstanding about gestational diet and weight management [38, 39]. A quantitative survey in Australia also revealed that pregnant women have insufficient knowledge of the Institute of Medicine gestational weight gain guidelines and pregnancy-specific dietary recommendations [40]. Knowledge is essential in the formation and change of behavior. Behavioral theories have shown that good attitude which transforms into good behavior can only be achieved when someone has adequate knowledge [41]. Furthermore, the overall effects, from our results showed that information was the most important influencing factor. Therefore, interventions to encourage pregnant women in undertaking weight management programs can focus on provision of adequate information.

A strong motivation is also required to facilitate the occurrence and maintenance of behavior aside adequate information and proficient behavioral skills [20], as it was observed in our study. GWM related motivation affects maintaining a healthy diet and appropriate physical activity during pregnancy. Previous works have confirmed the important role of motivation in weight management. The higher pregnant women awareness of their ideal pregnancy weight, the more likely their gestational weight gain be within the normal range [42]. Besides personal motivation, social motivation also had an influence on GWM behaviors. A study reported that maternity health professionals’ encouragement for pregnant women increased their GWM enthusiasm, and family support was very crucial [43]. Several studies highlighted the importance of motivational interventions for pregnant women. For instance, Hill et al. suggested that personal motivation is a key factor that can be considered when improving pregnant women’s dietary and physical activity behavior [44]; Furness et al. pointed out that motivation and social support are important factors for weight management, and interventions should focus on motivation strategies and social support facilitation [45]. Therefore, the maternal motivation can be enhanced from two aspects: strengthening the personal motivation and social motivation concerning to GWM.

Our findings also found the relationship between behavioral skills and health behaviors. Although maintaining a reasonable diet and exercise are the most effective ways to control gestational weight, it involves series of complex behaviors. Previous studies have shown that pregnant women had good understanding of pregnancy nutrition guidelines, but usually lacked the confidence and ability to put them into practice [46]. In terms of exercise, studies have also shown that pregnant women are not clear about which physical activity they can perform during pregnancy, as well as the frequency and intensity of the activity [28]. These uncertainties hinder their ability to perform physical activity. Studies have also reported that pregnant women often lack the ability to assess whether their weight gain is appropriate [47], which is not conducive for them to develop rational weight management strategies. To the best of our knowledge, few studies have validated the association between behavioral skills and GWM behaviors through a quantitative study, however, our results indicated that mastering more behavioral skills is a facilitator for weight management. Hence, in collaboration with previous qualitative researches, interventions can focus on training and guidance of pregnant women's behavioral skills.

### Strengths and limitations

A hypothetical model to predict gestational weight management was proposed based on IMB model for the first time, and was successfully verified through our empirical study. On the one hand, our research considered the influencing factors and mechanism of weight management during pregnancy with a more comprehensive perspective. On the other hand, it also promoted the application of the IMB model in the field of GWM. Nevertheless, this study also had some limitations: First, the cross-sectional study limits the causal inference of our findings, and longitudinal studies were needed for further verification. In addition, "stages of gestation" can be considered as a variable to construct a more rigorous and dynamic IMB model of gestational weight management. Second, although we considered measuring content of various factors as much as possible when designing the questionnaire, IMB model is rarely used in GWM studies, hence, the evaluating items might not be comprehensive [48]. Therefore, in the future, qualitative studies in the field of weight management during pregnancy can be carried out based on this theory, as well as reviewing similar studies, to explore a more comprehensive and accurate scale. It is recommended that interventionists investigate and understand the current status of information, motivation and behavioral skills among pregnant women before applying this model for maternal weight management, and then carry out targeted intervention guidance. Third, we employed a purposeful sampling method, and we selected pregnant women from only one hospital to validate the hypothesized model. Therefore, there may be selection bias. Forth, as this study used a self-administered questionnaire, information bias exists.

## Conclusions

Results indicate the gestational weight management behavior model which developed from IMB can be used as a theoretical guidance and intervention framework to carry out weight management in pregnant women. Information, motivation, and behavioral skills were all necessary factors and should be paid attention to simultaneously in weight management during pregnancy.

## Data Availability

Data are available upon reasonable request from the corresponding author. E-mail: pengxueqing1230@163.com.

## Abbreviations

IMB: Information-Motivation-Behavioral skills model
GWM: Gestational weight management
SEM: Structural equation modelling
BMI: Body mass index
KMO: Kaiser–Meyer–Olkin
CR: Composite Reliability
AVE: Average Variance Extracted
HTMT: Heterotrait-Monotrait
GOF: Goodness of fit
PLS-MGA: Partial least square multi-group analysis
In: Information
Mo: Motivation
BS: Behavioral skills
